# Novel concepts in cervical cancer screening: a comparison of VIA, HPV DNA test and p16^INK4a^/Ki-67 dual stain cytology in Western Kenya

**DOI:** 10.1186/s13027-020-00323-6

**Published:** 2020-10-02

**Authors:** Elkanah Omenge Orang’o, Edwin Were, Oliver Rode, Kapten Muthoka, Michael Byczkowski, Heike Sartor, Davy Vanden Broeck, Dietmar Schmidt, Miriam Reuschenbach, Magnus von Knebel Doeberitz, Hermann Bussmann

**Affiliations:** 1 Academic Model Providing Access to Healthcare (AMPATH), Eldoret, Kenya; 2 Department of Reproductive Health, School of Medicine, College of Health Sciences, Moi University, Eldoret, Kenya; 3 Applied Tumor Biology, Institute of Pathology, Heidelberg University Hospital, Heidelberg, Germany; 4 SAP SE, Walldorf, Germany; 5 International Centre of Reproductive Health, Ghent University, Ghent, Belgium; 6 National Reference Centre for HPV, Brussels, Belgium; 7 Laboratory of Molecular Pathology, AML, Antwerp, Belgium; 8 MVZ of Histology, Cytology and Molecular Diagnostics, Department of Cytopathology, Trier, Germany

**Keywords:** Cervical cancer screening, VIA, HPV, p16^INK4a^/Ki-67, LMICs, Dual staining, HPV genotype, HIV

## Abstract

**Background:**

Screening of unvaccinated women remains essential to mitigate the high morbidity/mortality of cervical cancer. Here, we compared visual inspection with acetic acid (VIA), recommended by WHO as the most cost-effective screening approach in LMICs, with HPV-based screening, and usage of p16^INK4a^/Ki-67 dual stain cytology.

**Methods:**

We prospectively enrolled women participating in a VIA-based cervical cancer screening program in two peri-urban health centers of Kenya. Consenting women had a VIA examination preceded by collection of a liquid-based cytology sample from the cervix stored in PreservCyt medium (Hologic®). Analysis of all samples included a hrHPV DNA test and evaluation of a p16^INK4a^ /Ki-67 (CINtecPLUS®) dual stained slide that was prepared using the ThinPrep® 2000 Processor and evaluated by a pathologist trained in the methodology.

**Results:**

In 701 of a total of 800 women aged 18–64 years, all three investigations were performed and data could be analyzed. The HPV, VIA and dual stain cytology positivity were 33%, 7%, and 2% respectively. The HPV positivity rate of VIA positive cases was 32%. The five most common HPV types were HPV16, 52, 68, 58 and 35. The OR among HIV infected women of an HPV infection, VIA positivity and positive dual stain cytology were 2.6 (95%CI 1.5–4.3), 1.9 (95%CI 0.89–4.4) and 3.4 (95%CI 1.07–10.9) respectively. The sensitivity of VIA to detect a p16^INK4a^/Ki-67 positive transforming infection was 13% (95%CI 2–38).

**Conclusions:**

Primary HPV testing appears feasible and should be considered as a primary screening test also in LMICs. The poor sensitivity of VIA renders it unsuitable as a triage test for HPV positive women. The utility of p16^INK4a^/Ki-67 dual stain cytology as a triage test for HPV positive women in LMICs should be further studied.

## Introduction

Cancer of the uterine cervix (cervical cancer) is the leading cancer in women in Sub-Sahara Africa [1]. New cervical cancer cases can be effectively reduced by screening tests that allow for the early detection and subsequent treatment of pre-cancerous lesions [2] and by vaccination against human papillomavirus (HPV) infection [3]. In parallel to the desired rapid scale-up of vaccination programs, screening remains important for both the vaccinated and especially the un-vaccinated population [4, 5].

Historically, the most impressive screening results were achieved in industrialized countries by regular cytological assessment of the cervix using the Pap test [6, 7]. However, this test is not considered suitable for screening in developing countries as it requires highly specialized personnel for evaluation and a reliable infrastructure for regular retesting due to the limited specificity and sensitivity profiles and high rates of equivocal results of the Pap test [8, 9].

Instead, for low-income countries the visual inspection of the cervix after application of acetic acid (VIA) coupled with subsequent management of abnormalities by cryotherapy is recommended, in what is now commonly referred to as “screen and treat” strategy [10–16]. VIA has the advantage of being inexpensive with a limited supply-chain burden and of providing results that are apparent at the time of the examination. However, VIA findings are not reliably reproducible and its accuracy for the identification of precancerous lesions is only moderate [17, 18]. Also, VIA programs have faced significant scale-up challenges [19, 20].

A hrHPV infection is a necessary factor for cervical cancer development [2, 21] and a negative HPV test provides high reassurance against precancerous lesions for at least 5–10 years [22]. The high sensitivity of the HPV DNA test [23–25] comes with the cost of moderate specificity as only a fraction of HPV infections progress to cervical cancer [2, 26].

To eliminate unnecessary follow-up of HPV positive women triage by a more specific test [27] is required. Among the novel, more disease-specific molecular markers of cervical cancer p16 ^INK4a^/Ki-67 dual stain cytology has been most extensively studied. This immunocytochemistry test detects cells that have undergone neoplastic transformation in the course of a persistent HPV infection, a stage in the HPV-mediated cervical carcinogenesis that is also called a transforming infection and that correlates morphologically with a CIN2/3 lesion [28–30]. Its high specificity in the diagnosis of cervical intraepithelial neoplasia of grade 2 or higher (CIN2+) has been demonstrated in large organized screening programs [31] and has been widely approved as a triage test for HPV-infected women.

The objective of the present study was to investigate the overall positivity rates for HPV and p16^INK4a^ /Ki-67 dual stain cytology in a peri-urban screening population of Kenyan women and compare the results with the performance of VIA.

## Patients and methods

### Study design and description of participants

We carried out a cross-sectional diagnostic study in two representative peri-urban health centers (Huruma and Uasin-Gishu) in Eldoret, Uasin Gishu County, Kenya. Women were recruited at the family planning clinics in both health centers. A total of 800 women were consecutively enrolled in this study between September 2016 to November 2017.

Women aged 18–64 years who were living in the catchment area of one of the recruiting health centers were candidates for the study. Eligibility included past or current sexual activity, an intact uterus, ability to undergo informed consent, an interview procedure, and a pelvic examination. Exclusion criteria included hysterectomy, history of cervical cancer, or current pregnancy. Nurses in the two health facilities identified potential participants who attended the clinic and explained the study in detail. Written informed consent was obtained in Kiswahili or English before study enrollment.

The clinical examination involved a gynecological examination with inspection of the cervix uteri and specimen collection by a trained female nurse in a separate room in the health centers. The study nurses in our two sites were trainer within the regional VIA screening program and thus well experienced in VIA examinations.

A cervical smear sample was collected for HPV DNA testing and p16 ^INK4a^ /Ki-67 dual stain cytology in PreservCyt® Solution (Hologic) using the Cervex-Brush® (Rover). The sample was then stored at ambient temperature (no direct sunlight) until tested. Finally, the cervix was evaluated 90 s after the application of 5% acetic acid (VIA), the findings were documented with a digital camera.

Women were informed about the VIA result immediately and told that they would get their laboratory test results within 2 months if they were positive. The relaying of laboratory results to the study participants was done through the established infrastructure by health extension workers who had a mobile phone and knew the women. Treatment was provided at the referral center.

### Laboratory testing

The first batch of HPV tests (*N* = 402) were done at the BIOZeq laboratory, Nairobi, Kenya, using the commercially available Hybrid Capture® 2 HR-HPV test (HC2) by Qiagen. The second batch (*N* = 383) was tested at AML laboratory, Antwerp, Belgium, using the TaqMan-based qPCR assay (RIATOL, Sonic Healthcare Benelux, Antwerp, Belgium) which detects 17 HPV genotypes and β -globin in seven multiplex reactions. These HPV types include all 12 high-risk types (HPV16, 18, 31, 33, 35, 39, 45, 51, 52, 56, 58, 59), three probably high-risk types (HPV53, 66 and 68), one low-risk type (HPV6) and one undetermined risk type (HPV67) [32]. All HC2 positive and a subset of HC2 negatives samples were retested using the TaqMan-based qPCR assay.

All dual stain cytology tests were done at the Department of Applied Tumor Biology, University Hospital Heidelberg (ATB), using CINtec PLUS p16/Ki-67® by Roche MTM Laboratories according to the manufacturer instructions. All slides were evaluated by a specialist pathologist. According to the manufacturer’s instruction, one double-stained cell was sufficient to score the sample as positive.

### Data collection

At enrollment information was elicited from participants on socio-demographic status and relevant sexual and reproductive health issues including HIV and ART status.

All study information was collected electronically except consent forms. A paper copy of the informed consent forms was offered to the participants. The consent forms with participants’ signatures and national ID numbers was collected and, at the end of the day, secured in locked file cabinets at the base sites. Labels for laboratory samples were handwritten and contained a computer-generated subject identifier and sample date. No personal identifying information can be derived from the labels.

The electronic database was designed in conjunction with SAP®, Walldorf, and consisted of six user interphases (recruiter, nurse, HPV and dual staining laboratory technician as well as HPV and dual stain cytology evaluator). Data captured by nurse, laboratory technician and gynecologist was pseudonymised and regularly uploaded to the HANAcloud®. Two-level passwords were required to perform computer entry and operate the networking programs.

### Statistical analysis

Continuous variables were summarized using mean and standard deviation. Categorical variables were summarized using percentages. Prevalence of HPV positivity, dual stain cytology positivity and of VIA positive results were calculated overall and by age groups. A chi-square test was used to compare proportions. Type-specific HPV prevalence was expressed as the proportion of women positive for a given HPV type among all women tested in the indicated group. Odds ratio and 95% confidence intervals were used to compare the within-group proportions. Logistic regression was used to estimate associations between risk factors and outcome of each of the three screening tests. All analyses were performed with SAS [computer program] Version 9.4. Cary, NC: SAS Institute Inc.. For Kappa agreements GraphPad was used (https://www.graphpad.com/quickcalcs/kappa2/. Accessed 15 December 2019).

### Ethics considerations

Study approval was granted from the local review board at Moi Teaching Referral Hospital (MTRH) and Moi University, Eldoret, Kenya, and the Institutional Review Board of the Heidelberg University Hospital, Germany.

## Results

### Study population

Of the 800 enrolled women, 701 (87.6%) had all three tests (VIA, HPV, and dual stain cytology) available for evaluation The baseline characteristics are summarized in Table 1. The median age (IQR) was 30 [25, 33] years. Sixty-four women reported their HIV status as positive, 625 as negative while 12 did not know their status.

**Table 1:** Baseline characteristics of study population

Variables	
Age in years	
Age, median (IQR)	30 (25,36)
Age < 30, n (%)	364 (53)
Age 30+, n (%)	323 (47)
Parity, n (%)	
1–4	593 (85)
More	64 (9)
None	44 (6)
Contraception, n (%)	
Contraceptive Injection	220 (43)
Intrauterine Contraceptive Device (IUCD)	122 (24)
Contraceptive Implant	99 (19)
Oral Contraception (OC)	61 (12)
Bilateral Tubal Ligation (BTL)	8 (2)
Condom	5 (1)

### Positivity rate of screening tests

**Fig. 1:**
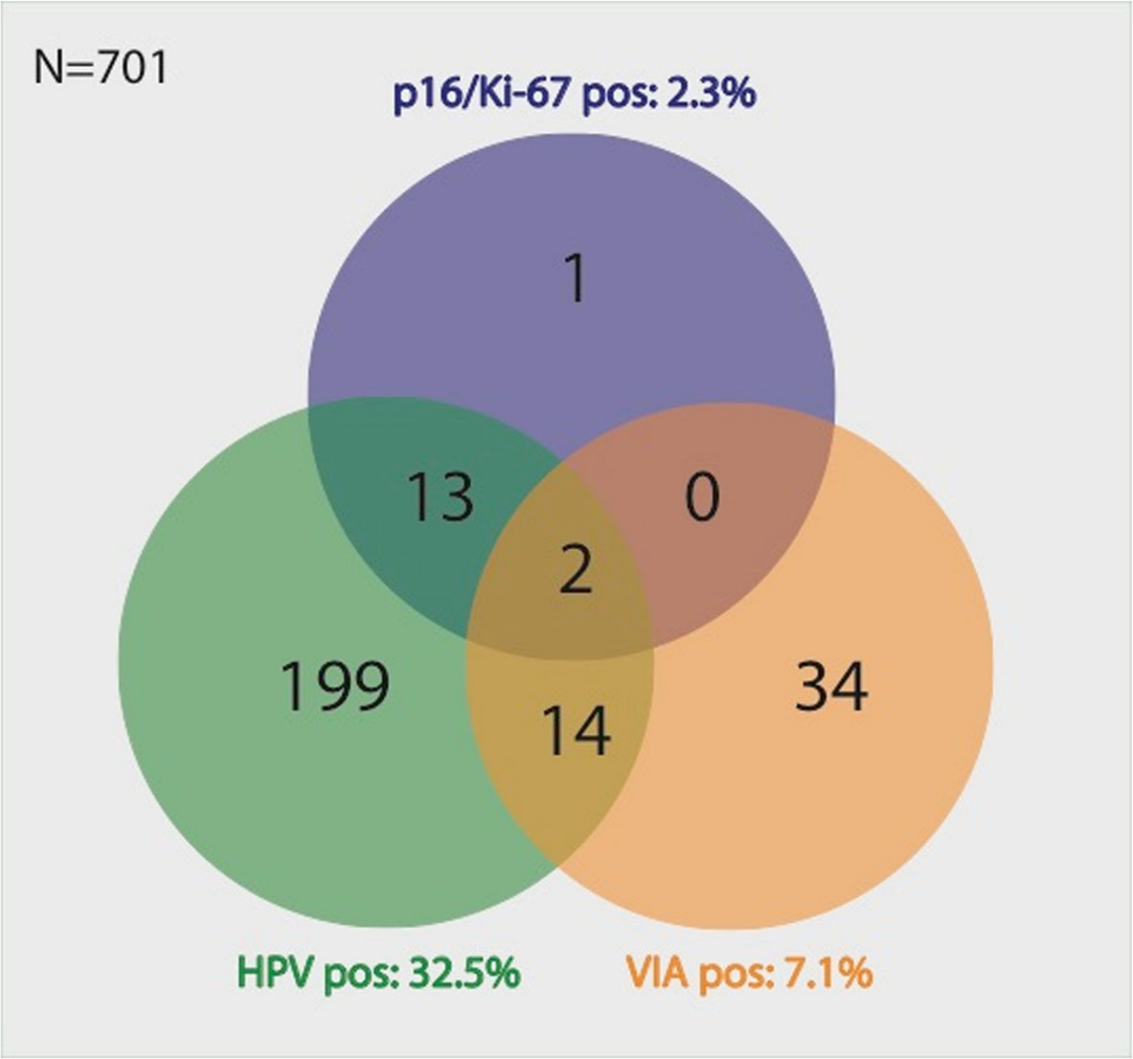
Relationship between test positivities of HPV test, VIA, and dual stain cytology

The overall test positivity of HPV, VIA and dual stain cytology were 32.5%, 7.1%, and 2.3% respectively (Table 2). Table 2 also depicts test positivity broken down in age groups. The relationship between test-positives is depicted as Venn diagram in Fig. 1. All 3 tests were positive in 2 cases, the HPV test was positive in 15 of 16 (93.8%) dual stain positive cases, the VIA was positive in 2 of 16 (12.5%) dual stain positive cases and HPV was positive in 16 of 50 (32%) VIA positive cases. The agreement between HPV status and VIA diagnosis had a Kappa of − 0.002 (95%CI − 0.053 to + 0.049).

**Table 2:** Prevalence of positive test results for VIA, p16 ^INK4a^/Ki-67 dual-stained cytology, and human papillomavirus testing ^*^p = 0.059 ^$^non-significant

	VIA positive			Dual-stain cytology positive			HPV positive		
Age group	No.	(%)		No.	(%)		No.	(%)	
all women (*N* = 701)	50	7,1		16	2,3		228	32,6	
women 18–29 y (*N* = 378)	23	6,1	**$**	7	1,9	**$**	137	36,2	*
women 30 + y (*N* = 323)	27	8,4		9	2,8		91	28,2

### Risk factors for a positive screening test

In logistic regression models including the risk factors ‘age over 30 years’, ‘HIV-infection’, ‘multipara’, ‘hormonal contraception’ and ‘multiple partners’ a significant association was found between HPV infection and HIV status (*p* = 0.0002), age (*p* = 0.023) and multiparity (*p* = 0.029) and between positive dual stain cytology and HIV status (*p* = 0.029). No significant association was found between positive VIA and any of the above risk factors. The odds of an HPV infection among HIV positives were 2.6 times higher than among HIV neg (95%CI 1.5–4.3). The odds of a positive VIA among HIV positives were 1.9 times higher than among HIV neg (95%CI 0.89–4.4). The odds of a positive dual stain cytology test among HIV positives were 3.4 times higher than among HIV negatives (95% CI 1.07–10.9).

### Frequency and distribution of HPV genotypes

**Fig. 2:**
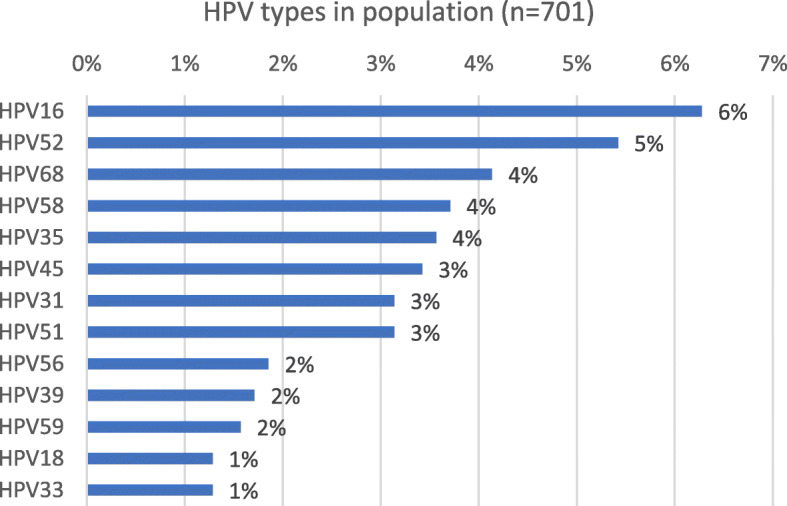
Type-specific HPV frequency in the study population (single and multiple infections, *N* = 284)

The type-specific hrHPV distribution of the study population is depicted in Fig. 2. The 5 most frequent genotypes were HPV16, 52, 68, 58 and 35.

**Fig. 3:**
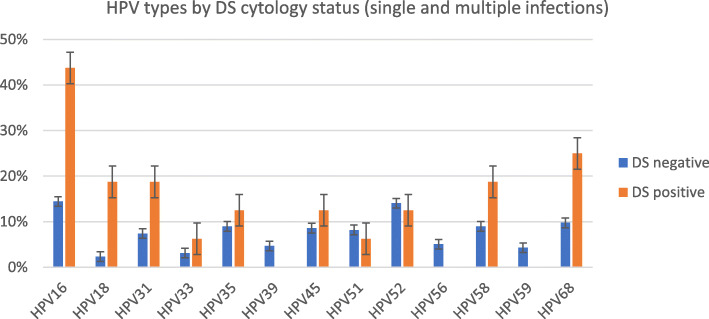
Proportion of HPV types among dual stain (DS) cytology positive (*N* = 15) and negative (*N* = 686) samples. Error bars represent standard errors

The frequency of hrHPV types among dual stain cytology positive samples is depicted in Fig. 3 and the odds ratio and corresponding 95% confidence intervals for the prevalence of the hrHPV types in dual stain positives compared with those of dual stain negatives are presented in Table 3. The odds ratio for HPV16, 18 in dual stain positive samples were highly significant. The 5 most common hrHPV types among dual stain positive cases were HPV16, 18, 31, 58, 68.

**Table 3:** Odds ratio and corresponding 95%CI for HPV types in dual-stain positive compared to dual-stain negative cases and in HIV positive compared to HIV negative cases, (* significant)

	Dual-stain cytology	HIV status
HPV 16	* 13.6 (95%CI 4.8–38.6)	0,7 (95%CI 0.2–2.3)
HPV 18	* 26.1 (95%CI 5.8–115.9)	1.2 (95%CI 0.15–9.95)
HPV 31	*8.1 (95%CI 2.1–30.8)	*3 (95%CI 1.1–8.5)
HPV 33	5,6 (95%CI 0.66–48)	*5.1 (95%CI 1.2–20.8)
HPV 35	4.1 (95%CI 0.9–19.1)	*5.1 (95%CI 2.1–12.4)
HPV 39	< 0.00	3.8 (95%CI 0.99–14.7)
HPV 45	4.3 (95%CI 0.92–20.1)	2,7 (95%CI 0.97–7.5)
HPV 51	2,1 (95%CI 0.3–16.7)	*3 (95%CI 1.1–8.5)
HPV 52	2.6 (95%CI 0.6–11.18)	*2.4 (95%CI 1.03–5.8)
HPV 56	< 0.00	< 0.00
HPV 58	*6.6 (95%CI 1.8–24.9)	2 (95%CI 0.7–6)
HPV 59	< 0.00	*4.3 (95%CI 1.1–17.2)
HPV 68	*8 (95%CI 2.7–29.2)	2.3 (95%CI 0.8–6.3)

The odds ratio and corresponding 95%CI of hrHPV types in HIV positives as compared with HIV negatives are presented in Table 3. HPV 31, 33, 35, 51, 52, 59, 68 were significantly more frequent in HIV infected women. HIV infected women also harboured more multiple hrHPV infections.

### Accuracy of HPV and VIA to detect a transforming HPV infection

The accuracy of VIA and HPV-testing among all women in detecting a p16 ^INK4a^ /Ki-67 positive transforming infection are shown in Table 4. HPV had a sensitivity of 0.94 (95%CI: 0.7–0.99) and a specificity of 0.69% (95% CI:0.65–0.72), PPV 0.07 (95% CI:0.06–0.08) and a NPV 0.99 (95% CI: 0.986–0.999). VIA had a sensitivity of 0.13 (95% CI:0.02–0.38), specificity 0.93 (95% CI:0.91–0.95), PPV 0.04 (95% CI:0.001–0.14) and a NPV 0.98 (95% CI,0.97–0.98). No significant difference was found between the age group less than 30 and 30+ years.

**Table 4:** Accuracy of HPV and VIA to predict p16 ^INK4a^/Ki-67 dual stain positive infections. PPV = positive predictive value, NPV = negative predictive value

Test	Sensitivity, % (95% CI)	Specificity, % (95% CI)	PPV, % (95% CI)	NPV, % (95% CI)
HPV	94 (70–100)	69 (65–72)	7 (6–8)	99 (99–100)
VIA	13 (2–40)	93 (91–95)	4 (1–14)	98 (97–98)
VIA triage of HPV positives	13 (1–40)	93 (89–96)	12 (3–36)	93 (92–95)

### Triage of HPV positives using VIA

Sequential testing i.e. VIA on HPV positive cases showed unchanged VIA sensitivity (0.13 (95% CI:0.01–40.5)) and specificity (0.93 (95% CI:0.89–0.96)), the PPV (0.12 (95% CI:0.03–0.36)) performed slightly better but the negative predictive value (0.93 (95% CI:0.92–0.95)) fared worse (Table 4).

## Discussion

### Main findings

Our study found a high burden of high-risk HPV infections among women attending family planning services in Western Kenya with a more than 2-fold higher burden among HIV infected women. VIA, the current screening test in Kenya and most sub-Saharan African countries (SSA), only poorly correlated with the HPV test, as nearly 2/3 of VIA positive women had no HPV infection and would thus be overtreated.

We used p16^INK4a^/Ki-67 dual stain cytology in order to differentiate common, and often spontaneously clearing HPV infections from persistent, transforming infections, and their morphological correlate, a CIN2+ lesions. When comparing VIA with dual stain cytology we found that VIA only detected a small fraction of transforming HPV infections and only few among them were HPV 16 and/or 18 infections, which have the highest potential to develop cancer.

A primary HPV testing strategy should be considered also in resource-poor countries. Provided its cost-effectiveness is established, dual stain cytology could be a suitable triage test. The assistance of an electronic data system will greatly facilitate such a multi-contact approach.

### Interpretations of results

#### HPV burden

We found a high overall burden (32.5%) of high-risk HPV infections in our study population, similar to results from another study in our Western Kenya region [34]. A number of other studies done in SSA report lower [33, 35–39] or similarly high [40, 41] HPV burden. All these studies were facility-based with often unknown cervical morbidity and different distribution of risk factors for HPV acquisition, i.e. enrolled populations differed by age distributions and HIV status. A true population HPV prevalence is still lacking for the region. Consistent among all of these studies was the 1.5–2 fold increase of HPV infection among the HIV infected as compared to HIV-negative women- when reported.

#### HPV genotypes

The five most common genotypes in our study were HPV16, 52, 68, 58, 35. Again, the genotype distribution is influenced by the state of the cervical disease, the geographic region as well as by technical issues including the HPV platform used, assay cut-offs and the selection of hrHPV types included in the analysis. In our study we were able to classify the genotype distribution according to the dual stain cytology findings. A strong association of HPV16, 18 and also HPV31, 58, 68 was found with dual stain cytology positive cases consistent with findings of a meta-analysis that compared normal and HSIL cases in East Africa [42]. In the dual stain negative group HPV16, 52, 35, 58, 68 were most common which is in line with a meta-analysis among African women with normal cytology [43, 44].

Surprisingly we found a high burden of HPV68, a genotype that is uncommon in epidemiological surveys worldwide. In our study the high proportion of HPV68 was found both in the dual stain positive and dual stain negative cases. Two-third of HPV68 was associated with multiple infections and its frequency was more than 2 times higher in HIV infected women. This is consistent with another study in the same region of Kenya where a high HPV68 prevalence among HIV-infected women was found [34]. A study conducted in an isolated rural community in Brazil reported HPV68 as the most prevalent genotype, however it was not present in women with cytological abnormalities [45]. The epidemiological importance of HPV68 needs to be further evaluated, especially as assay-related variation in HPV68 detection has been reported [46].

#### VIA

The overall VIA positivity in our study population (7.1%), diagnosed by well trained and experienced VIA nurses was similar to reports from India [47], Cameroon [39] and Tanzania [48] but lower than in a meta-analysis of 15 studies in SSA [49] were the pooled estimate of positivity was 17.4% (95&CI 10.4 to 25.6). A high variation in test positivity is observed among VIA studies conducted world wide [49], such discrepancies in VIA positivity is explained by patient characteristics (age, HIV status, precancer prevalence) and inherent procedure issues (high inter-operator variability, unamenable to quality control).

#### Dual stain cytology

The dual stain cytology positivity of 1.9% in the HIV uninfected population reflects the expected proportion of CIN2+ lesions in similar unscreened populations [49, 50]. Also, the strong association of the highly oncogenic HPV16, 18, 31 with dual stain positivity is well in line with the increased risk of (pre-)cancer associated with these HPV types [51].

All but one dual stain positive samples were also HPV positive. Possible explanation for such rarely found case [52–54] could be a low viral load or an HPV type not included in the common tests.

#### HIV/HPV co-infection

The detrimental effect of an HIV /HPV co-infection is well known [55, 56]. HIV significantly impacted on the outcome of VIA and dual stain cytology screening. The proportion of dual stain positivity was three times higher among the HIV infected population. The VIA positivity among the HIV infected women was twice as high (12.5%) compared to HIV uninfected women (6.7%). Studies of cohorts with high HIV burden in South Africa and Western Kenya reported high VIA positivity rates ranging from 22 to 55% [13, 57–59].

HIV-infected women in our study carried significantly more single and multiple hrHPV types compared to HIV-negative women. Among the currently available HPV vaccines the nonavalent (9v) vaccine would provide additional protection. Given the substantial number of non-9v hrHPV infections among HIV-infected women found by us and others [60, 61], however, careful post-vaccination followup will be important.

#### Evaluation of screening technologies

When comparing the studied screening techniques, the poor agreement between HPV status and VIA diagnosis is striking. Only 32% (16/50) of VIA positive cases were hrHPV positive or two third of women would be overtreated based on the VIA test result alone which is consistent with a study from Cameroon where half of all VIA/VILI-DC positive women had no associated hrHPV infection [39].

Our complete dataset gave us the opportunity to evaluate the performance of VIA and HPV testing when using dual stain cytology as a surrogate marker of high-grade cervical lesions. The dual stain biomarker p16^INK4a^/Ki-67 has a well documented high sensitivity and specificity for identifying the presence of (pre-)cancer [54, 62]. The low sensitivity of VIA in detecting a p16^INK4a^/Ki-67 positive transforming infections (Table 4) casts serious doubts on the use of VIA as a primary screening test.

As primary HPV testing will likely become the standard of care for cervical cancer screening in both low- and high-resource settings, the search for a suitable triage strategy to avoid overtreatment is crucial [27, 63–65]. The relatively high specificity of VIA suggests that VIA could be used as triage test for HPV positive women [66, 67]. In our study such approach would have slightly improved the positive predictive value of VIA but still be burdened with a low sensitivity.

Dual stain cytology is currently the most extensively studied molecular triage test [31, 68–70], that shows superior performance compared to other disease markers [71, 72]. In centralized laboratories the assay is performed on automated platforms offering high sample throughput. Recent advances in digital imaging and machine learning show promise to also automate the slide evaluation [73]. Although dual stain cytology is unsuitable for self-collected samples [74], it is worthwhile to evaluate the test as part of novel screening algorithms also in LMICS [75–77].

### Limitations

There were some limitations to this study. p16^INK4a^/Ki-67 is a very strong but not a definitive predictor of malignant degeneration (high-grade dysplasia). The gold standard for the diagnosis of high-grade cervical dysplasia is a cervical biopsy. Unfortunately, we could not perform colposcopies and biopsies for case ascertainment. Also, we used two different HPV DNA tests in our study, a nucleic acid hybridization test [78] and a PCR-based (GP5+/6+) test. Both tests, however, were validated in the Valgent study [78]. Finally, the facility-based recruitment, the rural setting and the relatively low sample size preclude generalization of the study findings.

## Conclusions

Our findings underscore the superior performance of HPV-based cervical cancer screening over VIA screening in detecting disease at-risk women. When used as triage of HPV positive cases in our high-prevalence setting the positive predictive value of VIA improved, but remained still flawed by an unacceptably low sensitivity. HPV testing should be considered as a primary screening test also in LMICs. P16^INK4a^/Ki-67 dual stain cytology is an excellent triage test for HPV positive women, its utility for LMICs needs to be further studied.

## Data Availability

The datasets used and/or analysed during the current study are available from the corresponding author on reasonable request.
